# Coping resources and stress due to demands in parents to children with autism spectrum disorder

**DOI:** 10.3389/fresc.2023.1240977

**Published:** 2023-10-06

**Authors:** Teresa Sartor, Sarah Sons, Jörg-Tobias Kuhn, Heinrich Tröster

**Affiliations:** Department of Rehabilitation Sciences, TU Dortmund University, Dortmund, Germany

**Keywords:** autism spectrum disorder, ASD, parental stress, parental demands, coping resources

## Abstract

**Introduction:**

Parents to children with autism spectrum disorder (ASD) are exposed to numerous demands in their daily lives and exhibit high levels of stress. The present study aims to find out which coping resources are mediators that help parents cope with these demands and which of those coping resources amplify or reduce stress arising from the demands. Studies often only focus on the connection between coping resources and stress without taking the demands into account at the same time.

**Methods:**

For this reason, a mediation model was set up to answer the research question. Data from a German questionnaire survey with *N* = 266 parents who have children with ASD (two to 23 years old) were used. Subjectively perceived demands in everyday life (scale “Parental demands in everyday life”), parental stress (“Parental Stress Inventory”, based on Abidin's parenting stress model) and the following coping resources were collected: parental self-efficacy beliefs (“Parents’ sense of competence questionnaire”), available social support of parents (scale “Availability of social support”) and parental coping strategies (German version of the Brief COPE).

**Results:**

An exploratory factor analysis revealed four mediators: dysfunctional coping, functional coping, social support, and self-efficacy. The use of dysfunctional behavior and parental self-efficacy were found to be significant mediators that mediated between daily demands and parental stress. A direct effect of demands on parental stress was also found, implying partial mediation. The two factors of functional coping and support were not found to be significant mediators.

**Discussion:**

Key findings indicate that parental stress resulting from the daily demands of parenting children with ASD can be reduced by high parental self-efficacy and increased by dysfunctional coping. For practice, it can be deduced that dysfunctional coping strategies of parents to children with ASD should be reduced and parental self-efficacy should be strengthened in order to reduce stress which arises from the multiple demands in everyday life.

## 1. Introduction

### 1.1. Demands and stress of parents to children with ASD

#### 1.1.1. Parental stress

Parents to children with autism spectrum disorders (ASD) exhibit higher levels of stress than other parents [e.g., (1–3)]. Based on the transactional stress model (4, 5), parental stress arises from the discrepancy between parents' demands and resources. Thus, it arises when parents perceive the resources available to them as inadequate for coping with the demands. Parents to children with ASD experience multiple demands in their daily lives (6), especially from the child's behavior and characteristics (7). In his parenting stress model, Abidin (8) calls this source of stress the child domain. This includes, among other stress sources, the inability to adapt to the environment [e.g., through restricted repetitive behavior of children with ASD; (9)]. These behavioral dispositions of the child result in special demands the parents are confronted with in their interaction with the child. In contrast, the parent domain reflects the stress resulting from the limitations of parental functions needed to cope with the educational demands. They include the restrictions on parents' personal lives that they experience as a result of their role as parents, or feelings of guilt when parents are not always emotionally available to their child (8). Results show that both domains are associated with psychovegetative stress symptoms and self-reported stress (10).

#### 1.1.2. Parental demands in everyday life

Day by day, parents to children with ASD have to cope with a variety of demands, which are associated with the role those parents take in different fields of everyday parenting (11). These parental demands result from the symptomatology of ASD [e.g., persistent deficits in social interaction or communication; (9)] as a profound developmental disorder and lead to impairments in almost all areas of life (12). These impairments create high demands on the caregivers, usually parents [e.g., (13)]. Tröster and Lange (6) have described the daily demands of parents to children with ASD in eight areas (see Table 1). The demand of organizing family life can be an extensive task with a child with ASD being a family member, as the child's disorder makes it particularly relevant for structures to be in place (14). A further demand is cooperating with the partner and maintaining the relationship (15) as well as keeping and strengthening the parent-child relationship (16). In addition, there are demands to maintain a social life (17) but also to fulfill one's own needs (18). There is also a lot of organizing and communicating to do outside of the family's daily routine, such as with funding agencies, therapists, or childcare facilities (19). Furthermore, parents are often faced with stigmatizing reactions in the social environment which they have to deal with (20). Managing the problem behavior of the child with ASD [e.g., restricted repetitive behavior; (21)] may prove to be a permanent demand in everyday life (17). In the study by Tröster and Lange (6), problematic behavior of the child in education was shown to be the most important predictor of stress in the child domain for parents to children with ASD. In the parent domain, balancing personal needs and interests with parental demands was shown to be predictive of stress.

**Table 1:** Subscales of the “Parental demands in everyday life” scale.

Parental demands	Example item	*α*
Organization of family life (5 items)	*Joint family activities require a lot of preparation.*	.73
Professional support (2 items)	*There are disputes with cost bearers when it comes to covering the costs of supporting my child.*	.54
Social participation (2 items)	*I find little time to spend with my friends.*	.80
Cooperation with the partner (4 items)	*Coordinating with my partner about the tasks in raising and caring for our child is difficult.*	.79
Parent-child relationship (4 items)	*I find it difficult to put myself in my child's shoes.*	.69
Personal way of life (5 items)	*I have to give up things that I enjoy doing.*	.90
Stigmatizing reactions in the social environment (7 items)	*In our circle of acquaintances, I experience misconceptions and prejudices towards my child.*	.73
Problem behavior of the child in education (12 items)	*My child is hypersensitive or hyposensitive to certain stimuli.*	.75

Subscales of the “Parental demands in everyday life” scale (6) including a sample item and internal consistency (Cronbach's alpha) for each scale.

### 1.2. Resources for coping with stress

In order to prevent stress from occurring in the first place, resources are necessary that enable or facilitate coping with the demands of parenting. Three resources have shown to be essential: self-efficacy, social support, and coping strategies.

#### 1.2.1. Parental self-efficacy

Self-efficacy, according to Bandura (22), is a person's subjective conviction regarding his or her own competencies, meaning that a person is convinced that his or her own competencies are sufficient to cope with upcoming challenges. Educational self-efficacy can be seen as parents' conviction that they are able to cope with the demands of parenting in the best possible way (23). For parents to children with ASD, research indicates a negative relationship between self-efficacy and parenting stress [e.g., (6, 24–26)]. Strong parental self-efficacy among parents to children with ASD may positively affect coping with challenging ASD-typical demands (27) and could protect against the development of parental depression (25). Daulay et al. (28) findings show an association between the severity of stereotypical and social abnormalities of the child with ASD and their parents' self-efficacy. A comparative study by Smart (29) shows that parents to children with ASD in particular have low self-efficacy compared to other parents, for example parents to children with Down syndrome or emotional behavioral disorders, which can be used as an explanation for a high stress level among parents to children with ASD.

#### 1.2.2. Available social support

Available social support represents another resource. Social support fulfills three functions: the (1) informational, (2) emotional, and (3) instrumental support (30). Exchanges among parents (informational) about experiences, for example, can be profitable since they offer an opportunity to ask another parent for advice. Social support can also facilitate emotion regulation when there is someone to talk to about thoughts and feelings. One example of instrumental social support is having access to someone who will provide childcare if needed. Knowing that social support is available can reduce stress in childrearing [e.g,. (10)]. Stress can be prevented by parents' confidence that they will find support in their social network when they need it (31). Studies agree that available social support is an important resource for parents to children with ASD [e.g., (32–34)]. For example, Siman-Tov and Kaniel (35) use path analyses to show that social support facilitates coping with stress that arises from parenting a child with ASD.

#### 1.2.3. Coping strategies

In addition, there are different strategies to cope with demands and deal with stressful situations which can be either functional, i.e., conducive to health, or dysfunctional, i.e., hazardous for health (36). Functional strategies such as exercising or performing relaxation exercises have shown to be preventive for burnout syndrome (37), whereas withdrawing and playing computer games, for example, are cited as dysfunctional because they promote the occurrence of the above stress disorder. According to the systematic review by Vernhet and colleagues (38), unlike parents to neurotypically developing children, parents to children with ASD use less social support and are more likely to use avoidance strategies to cope with demands. For example, avoidance behaviors manifest themselves in distractions such as watching television or by parents waiting it out in hopes that the problem will go away (39). Studies show that those parents to children with ASD who use dysfunctional behaviors such as active avoidance of problems exhibit higher levels of stress [e.g., (6, 40)], whereas active coping has been shown to be a stress-reductive strategy among parents to children with ASD (41). In active coping behaviors, parents perceive the stress-inducing demands and attempt to change them by taking action to address them (42).

To our knowledge, there is no study which examined the relationship between all three of these within a single mediation model: daily demands, coping resources, and parenting stress of parents to children with ASD. The focus is either on relationships between demands and parental stress (see Section 1.1) or on relationships between coping resources and parental stress (see Section 1.2). In the present study, the three aforementioned areas will be examined together to determine whether stress of parents to children with ASD arises from demands, and whether parental self-efficacy, social support, and coping strategies have a mediating effect of the stress level resulting from those demands. It was hypothesized that self-efficacy, available social support as well as functional coping strategies have a relieving effect, whereas dysfunctional coping strategies are hypothesized to increase parental stress.

## 2. Methods

### 2.1. Analytic strategies

Various parental coping strategies, social support, and self-efficacy were considered as potential mediators. In a first step, an explorative factor analysis of the various potential mediators was carried out with the aim of combining them into factors (see Section 2.3). The cutoff value was set at a factor loading of .30 because Hair et al. (43, p. 129) defined a factor loading of .30–.40 as “minimally acceptable”. Subsequently, multiple mediation analyses were calculated, in which demands were entered as an independent variable and parental stress as a dependent variable. The mediation analyses were conducted using R version 4.1.1 (44) and the lavaan package (45). The comparative fit index (CFI; cutoff value close to .95), the Tucker-Lewis index (TLI; cutoff value close to .95) and the root mean square error of approximation (RMSEA; cutoff value close to .06) were used as fit indices (46).

### 2.2. Data collection and sample

The data stems from the German research project “ELKASS” [Parents to Children with Autism Spectrum Disorders; (6)], which was developed in cooperation with the German Autism Association (“Autismus Deutschland e.V., Bundesverband zur Förderung von Menschen mit Autismus”) and ten autism therapy centers throughout Germany. Data collection took place between December 2015 and March 2017 and was reviewed for compliance with ethical guidelines prior to commencement. All parents surveyed were fully informed of the voluntary nature, anonymity, and intended use of the data prior to their participation and signed the consent declaration. *N* = 266 parents to children with ASD whose children had just started support in an autism therapy center were surveyed. Thus, the inclusion criterion was that the parents and their child were affiliated to a cooperating autism therapy center. Further inclusion or exclusion criteria were not specified. The therapists distributed the paper-based questionnaire and collected it again in an anonymized letter and forwarded it to the research group, who entered the information digitally into a data set and processed it for the analyses. The questionnaire was in German. The parents who participated in the survey questionnaire were aged between 24 and 63 years (*M* = 41.4 years, *SD* = 7.47 years) and the majority were mothers (86.1%). Most of them were married (72.6%) and almost a quarter (23.7%) described themselves as single parents. Their children were predominantly male (83.2%) and were between two and 23 years old (*M* = 10.2 years, *SD* = 4.0 years). Half of the children (50%) were diagnosed with at least one comorbid disorder in addition to their ASD (see Table 2). Information on diagnoses was requested from the therapists. Despite the wide age range, in what follows, we refer to them as children because they are their parents' children regardless of age, and this group is the focus of this research.

**Table 2:** Diagnosis and comorbid disorders of children with ASD.

	*n*	%
Diagnosis		
Asperger syndrome (F84.5)	127	47.7
Childhood autism (F84.0)	83	31.2
Atypical autism (F84.1)	32	12.0
Other diagnosis^a^	16	6.0
Comorbid disorders (three most frequent)^b^		
Hyperkinetic disorder (F90.-)	47	17.7
Specific developmental disorder of speech and language (F80.-)	20	7.5
Specific developmental disorder of motor function (F82.-)	17	6.4

Diagnosis and the three most frequently cited comorbid disorders with ICD-10 codes (21) of *N* = 266 children (deviations due to missing values).

^a^ The remaining category (7.8%) included, e.g., Pervasive developmental disorder, unspecified (F84.9) or cases where no firm diagnosis had yet been made.

^b^ Other comorbid disorders included e.g. Other behavioral and emotional disorders with onset usually occurring in childhood and adolescence (F98.-) or Mixed specific developmental disorders (F83).

### 2.3. Materials

#### 2.3.1. Parental demands in everyday life

The construct of parental demands was assessed with the scale “Parental demands in everyday life” by Tröster and Lange (6). The scale comprises a total of 41 items, which can be assigned to eight areas of demand. The items contain typical statements that describe the demands of parents in their everyday lives. The parents rated these with a four-point scale (1 = “never/rarely” to 4 = “very often”), depending on how often they experience the described situation. The eight subscales, the number of items and the Cronbach's α are shown in Table 1. In addition, an example item can be found for each case.

#### 2.3.2. Parental stress

Parental stress was measured with two instruments: the “Parental Stress Inventory” (“Eltern-Belastungs-Inventar”; EBI) and the Symptom List “Physical Complaints” [both (10)]. The symptom list Physical Complaints, which comprises a total of 14 items, records psychovegetative stress symptoms. The parents indicated on a five-point scale (1 = “never/almost never” to 5 = “very often”) how often they suffered from physical complaints, such as headaches/migraines, sleeping disorders or gastrointestinal complaints, in the past week. The scale showed good internal consistency of Cronbach's *α* = .87. The Parenting Stress Inventory is a screening instrument based on the parenting stress model by Abidin (47) that measures different sources of stress for parents (10). The inventory of 48 items overall is divided into two domains (see Table 3): The parent domain consists of seven subscales that capture the stress resulting from the tasks parents have to cope with in their role as parents. The child domain comprises five subscales of stress resulting from the child's characteristics and behavior. In the present investigation, Cronbach's α reliabilities in the two domains were high (parent domain: *α* = .93; child domain: *α* = .88). All items are answered on a five-point scale from 1 = “does not apply at all” to 5 = “fully applies”. The validity of the instrument was predominantly demonstrated with mothers of children and adolescents with disabilities and chronic illnesses (10).

**Table 3:** The parent and child domain subscales of the EBI.

	Example item	*α*
Parent domain		
Health	*I don't have as much energy anymore to do things that I used to enjoy.*	.85
Depression	*I sometimes feel like it's actually my fault when my child does something wrong.*	.81
Parental Attachment	*It sometimes takes a while for parents to develop a feeling of closeness and warmth for their child.*	.73
Parental Competence	*Some things in raising my child are harder for me than I expected.*	.81
Role Restriction	*I sometimes feel constrained by the responsibilities of being a mother/father.*	.85
Isolation	*I don't have as much interest in other people as I used to.*	.76
Spouse	*Since I had the child, I don't do as much together with my partner.*	.80
Child domain		
Demandingness	*I have the impression that my child needs more attention and care than other children.*	.75
Acceptability	*I sometimes have the impression that my child is not as open to other people as other children.*	.52
Adaptability	*My child often reacts very strongly when something happens that he doesn't like.*	.78
Distractibility/Hyperactivity	*I often feel exhausted because my child is so active.*	.71
Mood	*My child often wakes up already in a bad mood.*	.80

Presented are the subscales of the EBI (10), each with an example item and the internal consistency (Cronbach's alpha).

#### 2.3.3. Parental self-efficacy beliefs

Parents' self-efficacy beliefs regarding the effectiveness of parenting children with ASD were assessed using Miller's (48) “Parents’ sense of competence questionnaire”. The scale uses seven items to survey parents' sense of self-efficacy in raising their child (example item: “I am meeting my personal expectations about how I care for my child”). The scale was also answered by parents using a four-point scale ranging from 1 = “not true” to 4 = “completely true”. Cronbach's α in the present sample was *α* = .75.

#### 2.3.4. Available social support of parents

The scale “Availability of social support” (10) was used to survey available social support. The scale depicts how well parents perceive the availability of their social support, which is surveyed with nine items in three areas [(1) Informational support: “Through my circle of friends and acquaintances I often get good tips (e.g., good doctor, events)”; (2) Instrumental support: “I know some people I could ask for support if I were in financial need”; (3) Emotional support: “I have some good friends with whom I can talk about personal problems”]. Internal consistency was very good (Cronbach's *α* = .91). The response format of the items comprised four grades (1 = “not true” to 4 = “completely true”).

#### 2.3.5. Parental coping strategies

In order to survey different coping strategies, the German translation of the frequently used instrument Brief COPE (42); German translation by (49) was used. According to Carver (42), of the total 28 items, two items each are assigned to one of the 14 subscales, which represent different habitual coping strategies: denial (e.g., persuading oneself that the situation is not so bad), self-blame (e.g., blaming oneself for being responsible for the situation), self-distraction (e.g., occupying oneself with other things), venting emotions (e.g., showing others one's own feelings), substance use (e.g., drinking alcohol to feel better about oneself), positive reframing (e.g., looking at the positive side of a situation), planning (e.g., considering what would be the right thing to do in a situation), acceptance (e.g., accepting that certain things cannot be changed), active coping (e.g., being proactive to change the situation), humor (e.g., making jokes about the situation), use of emotional support (e.g., accepting consolation from other people), use of instrumental support (e.g., asking other people for advice), behavioral disengagement (e.g., not dealing with the situation), religious coping (e.g., finding support in one's own faith). This structure has been used previously in studies of parents to children with ASD (50). In the present study, a 15th subscale (“negative thoughts and reactions”) was used, which was developed as an addition by Tröster and Lange (6). The coping strategy “negative thoughts and reactions” also contains two items and represents the parents' negative thoughts resulting from the child's behavioral problems (example item: “I thought that my child was deliberately trying to provoke me.”). The items of the Brief COPE and the additional subscale are answered with a four-point scale (1 = “does not apply at all” to 4 = “applies very much”).

## 3. Results

### 3.1. Exploratory factor analysis

In order to assess the dimensionality of coping strategies, the structure of the subscales of the Brief COPE (42); surveyed with the German translation by (49) was examined by means of an exploratory factor analysis (principal component analysis with Promax rotation). The scale “availability of social support” (10) was included in the analysis due to its content-related fit. Knowing that there is social support of a network that one can fall back on in case of need may encourage parents and was thus regarded as a potential coping strategy.

A parallel analysis clearly showed three factors above the threshold in the principal component analysis. By means of the EFA, a total of 38.6% of the variance could be extracted on the basis of three factors with an eigenvalue >1 (see Table 4). The three factors that emerged were (1) dysfunctional coping, (2) functional coping and (3) support.

**Table 4:** Results of the exploratory factor analysis.

Coping strategies	Factor 1	Factor 2	Factor 3	*h* ^2^
Denial	.682			.410
Self-blame	.650			.421
Negative thoughts and reactions^a^	.636			.358
Self-distraction	.456			.276
Venting emotions	.419			.262
Substance use	.337			.144
Positive reframing		.753		.501
Planning		.649		.507
Acceptance		.559		.268
Active coping		.546		.366
Humor		.440		.180
Use of emotional support			.872	.824
Availability of social support			.599	.363
Use of instrumental support			.494	.529
Explained variance in %	13.8	14.0	10.8	

Factor loadings and communalities (*h*^2^) of the exploratory factor analysis with the subscales of the Brief COPE (42); surveyed with the German translation by (49) and the “Availability of social support” scale (10).

^a^ The subscale “Negative thoughts and reactions” is a development of Tröster and Lange (6).

#### 3.1.1. Factor 1: dysfunctional coping

The factor “dysfunctional coping” was shown by strategies such as denial, self-blame, negative thoughts, or substance use. Self-distraction could also be assigned here, which was probably used as a displacement strategy. The “venting” strategy, which was assigned to dysfunctional coping in the analysis, also included statements such as “I've been expressing my negative feelings”. Especially in child rearing, omitting negative moods towards the child can lead to feelings of guilt, so this strategy probably did not lead to a reduction in stress. The factor “dysfunctional coping” was therefore characterized by the fact that those parents to children with ASD had and gave free rein to negative feelings towards the child as well as towards themselves based on self-blame. They denied the stress they experienced and repressed it through distraction or alcohol and drugs. This factor accounted for 13.8% of the total variance.

#### 3.1.2. Factor 2: functional coping

The second factor “functional coping” consisted of the subscales positive reframing, planning, acceptance, active coping and humor (14% variance clarification). The parents who used these functional behaviors as coping strategies seemed to approach the demands placed on them actively, so that they positively reinterpreted the situation for themselves, dealt with it in detail or also accepted the situation as it was and made the best of it. Humor can be understood as a displacement mechanism, but it can also lighten up a situation so that it is not treated too seriously and doggedly (“I've been making fun of the situation”).

#### 3.1.3. Factor 3: social support

The third factor “social support” was characterized by three forms of support: emotional, instrumental, and available social support. Parents who applied these strategies for themselves used their social contacts to experience emotional support as well as practical support. They felt certain that they could theoretically receive support if they ever needed it. The subscale “behavioral disengagement” could not be included in the EFA due to unacceptable reliability (Cronbach's *α* = .20). The subscale “religious coping” showed loadings that were too small on the support factor (<.3), which is why it was not included in the factor.

### 3.2. Mediation analysis

The three coping strategies dysfunctional coping (*M* = 1.71, *SD *= 0.46), functional coping (*M* = 2.61, *SD* = 0.57) and social support (*M* = 2.34, *SD* = 0.71) as well as the resource self-efficacy (*M* = 2.97, *SD* = 0.44) were included in the analysis as a total of four mediators. In addition to the basic assumption that stress arises from the demands that cannot be met or are difficult to meet, it is assumed that the four mediators affect the influence of the demands on stress. It is assumed that the use of dysfunctional behavior to cope with demands makes it more difficult and thus leads to stress, and that the use of functional strategies as well as social support and a pronounced self-efficacy facilitate coping.

**Figure 1:**
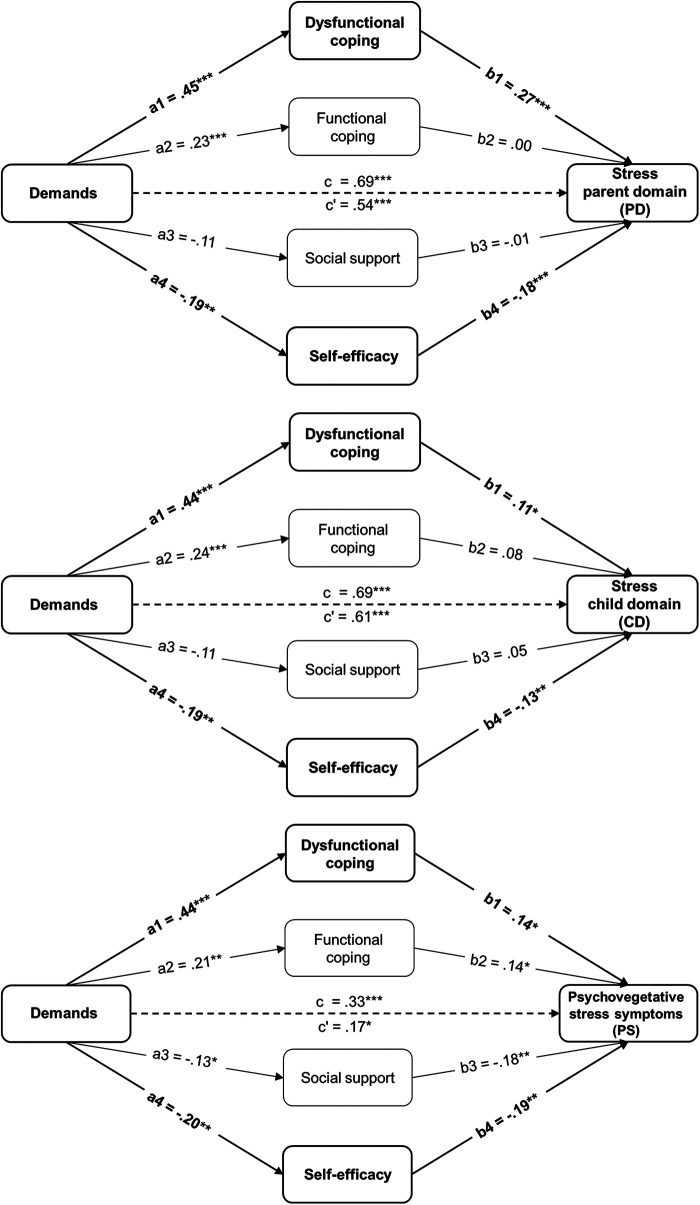
Results of the three mediation models. The three mediation models show demands as independent variable (IV), four mediators (dysfunctional coping, functional coping, social support, and self-efficacy), and stress as dependent variable (DV). The models differ in the domains of stress (parent domain, child domain, and psychovegetative stress symptoms). Path coefficients: c = total effect, c’ = direct effect, a = relationship between IV and mediator, b = relationship between mediator and DV. The significant indirect effects are shown in bold print. *p < .05, **p < .01, ***p < .001.

Three models are shown that differ only in the dependent variable. Here, stress is distinguished in the three facets of parent domain (PD), child domain (CD), and psychovegetative stress symptomatology (PS). Figure 1 presents the results of the mediation analyses. Model fit of the three models was good: PD: χ^2^ (15) = 410.22, *p* < .001, CFI = 1.0, TLI = 1.0, RMSEA = .00; CD: χ^2^ (15) = 389.92, *p* < .001, CFI = 1.0, TLI = 1.0, RMSEA = .00; PS: χ^2^ (15) = 248.79, *p* < .001, CFI = 1.0, TLI = 1.0, RMSEA = .00.

The direct path from demands to stress shows up significantly in all three models (c_PD _= .54, *p* < .001, *SE* = .080; c_CD_ = .61, *p* < .001, *SE* = .077; c_PS_ = .17, *p* < .001, *SE* = .011). Thus, there seems to be a direct influence of daily demands on stress across all domains. The total effect has higher path coefficients than the direct effect in all models, indicating partial mediation (c‘_PD_ = .69, *p* < .001, *SE* = .074; c‘_CD_ = .69, *p* < .001, *SE* = .067; c‘_PS_ = .33, *p* < .001, *SE* = .097). Partial mediation shows that stress in all three models arises largely from demands. Yet, it is also in part dependent on resources used. Indeed, it seems to be crucial for the stress of parents whether dysfunctional coping strategies are used to cope with the demands of everyday life (a1*b1_PD_ = .12, *p* < .001, *SE* = .042; a1*b1_CD_ = .05, *p *= .038, *SE *= .035; a1*b1_PS_ = .06, *p* = .043, *SE* = .049). The more demands occur, the more likely parents are to use dysfunctional behavior to cope, and this results in an increased occurrence of stress. The degree of parental self-efficacy also seems to mediate the effects of demands on stress to some extent (a4*b4_PD_ = .03, *p* = .018, *SE* = .023; a4*b4_CD_ = .03, *p* = .042, *SE* = .018; a4*b4_PS_ = .04, *p* = .028, *SE* = .029). When demands are high, self-efficacy decreases and, thus, stress increases. In terms of the resource, a pronounced self-efficacy provides relief. Engagement in functional coping (a2*b2_PD_ = .00, *p* = .976, *SE* = .019; a2*b2_CD_ = .02, *p* = .166, *SE* = .020; a2*b2_PS_ = .03, *p* = .083, *SE* = .027) and social support (a3*b3_PD_ = .00, *p* = .828, *SE* = .008; a3*b3_CD_ = -.01, *p* = .360, *SE* = .020; a3*b3_PS_ = .02, *p* = .108, *SE* = .023) do not appear to substantially mediate the effect of demands on experienced stress. Although in the model of self-reported psychovegetative stress symptomatology paths a and b each show significant coefficients for both mediators (see Figure 1), the indirect effect is not significant.

## 4. Discussion

The present study investigated the extent to which coping resources mediate the influence of everyday demands on stress experienced by parents to children with ASD. In the direct pathway as well as in the indirect pathways, there were no differences between the three dependent stress variables. Thus, the results of the models are discussed uniformly for the three stress domains surveyed (parent domain, child domain, psychovegetative stress symptoms) instead of separately.

The results confirm that the high demands of everyday life experienced by parents to children with ASD lead to stress. The direct effect shows that stress also arises from the demands independently of the coping resources collected in this study. According to transactional stress theory (4, 5), stress arises when there is a discrepancy between demands and perceived resources. It can be assumed that other coping resources that were not surveyed in this study should have been included as mediators. Possible mediators that studies have shown to be associated with parental stress include resilience [e.g., (51)] and sense of coherence [e.g., (52)].

The expectation that parental self-efficacy protects against stress arising from the demands placed on parents can be confirmed based on the results. The belief that they know best what exactly their child with ASD needs in certain situations [“If anyone knows the answer to what is wrong with my child, it is me”; (48)] and being able to meet these needs of their child seems to help parents cope with the demands of everyday life.

A larger mediation effect than in self-efficacy was shown for dysfunctional coping in the present models. Using dysfunctional coping strategies such as denial, self-blame, self-distraction, venting emotions, substance use, or negative thoughts makes it more difficult for parents to cope with the demands, which translates into increased stress levels. Dysfunctional strategies are not constructive or solution-oriented strategies. From ignoring the demand, as by denying, self-distraction or taking drugs, a demand (e.g., care of the relationship between parent and child, partnership or organization of family life, see Table 1) can be coped with only with difficulty. Stress arises as a consequence. It is also not helpful if parents get lost in negative thoughts and blame themselves.

Contrary to expectations based on studies indicating a link between functional coping and lower perceived stress (53), using a functional behavior to cope with demands does not appear to have any mediating influence on stress. Thus, parental stress arises from demands independent of the application of positive reframing, planning, acceptance, active coping, or humor. Possibly the functional strategies captured by the Brief COPE are not appropriate for the specific demands of parents with ASD children or they are too imprecise and would have to be adapted to typical situations in the everyday life of this target group.

In the study by Robinson and Weiss (33), both perceived and received social support were associated with lower stress among parents to children with ASD. The mediator “social support” in the present study was also composed of the two components of perceived social support (scale: Availability of social support) and received social support (subscales Brief COPE: Use of emotional support and Use of instrumental support). However, no effects could be shown regarding social support having an influence on whether stress arises from the demands of parents. For social support to be helpful in coping with demands, social contacts must be present and maintained. Studies show that parents to children with ASD, unlike other parents, use less often strategies to seek social support (38). In addition to the relieving function, the use of social support is associated with costs (54). Hierarchical structures that arise between the giver and the receiver of support can give rise to feelings of inferiority on the part of the receiver as well as feelings of inability, which can threaten self-worth (55). Feelings of obligation to return the support received or to be indebted to the other may also arise (56), which may discourage parents to children with ASD from seeking and accepting social support. Another explanation for the lack of effect in the present study is that such support, which would help parents cope with everyday demands, was not explicitly surveyed: for example, parents to children with ASD might benefit less from the opportunity to receive practical childcare support, because implementing this support requires first investing effort so that a child can be cared for by another person (e.g., practicing fixed routines with the child). Having another caregiver can also be stressful for the child, which in turn transfers to the parent. Parents would therefore likely benefit from long-term, dependable social support. To prevent feelings of imbalance, support groups could come together. Support groups are a good example here that this type of support can be helpful (57). Parents have the opportunity to support each other in an emotional way, but also to exchange information. Here they can share their experiences and feel self-efficacious by talking about issues they are familiar with: Parenting a child with ASD.

In practical work with families to children with ASD, parental self-efficacy should be strengthened in a professional setting using empirically tested interventions. For example, Sofronoff and Farbotko (58) evaluated an intervention program aimed at parents to children with Asperger's syndrome that had a demonstrable positive effect on the self-efficacy of parents and especially mothers. The intervention program is designed to increase parents' self-efficacy by improving their ability to better understand and manage their child's problem behavior. By better understanding towards their child's behaviors, they feel more confident in their response and more competent in their parenting role. A practical implication that would support this would be for parents to attend the child's therapy sessions. This way, the parents learn how to deal with the child's behavior and can apply this themselves to feel more confident and competent in everyday situations.

In summary, the diverse everyday demands of raising children with ASD lead to stress. The hypothesis that self-efficacy, social support, as well as functional coping strategies have a relieving effect on parents to children with ASD, while dysfunctional coping strategies increase parental stress, was partially confirmed. The daily demands are mainly influenced positively by parental self-efficacy and negatively by dysfunctional coping strategies. Above all, the practice should therefore strengthen the self-efficacy of parents to children with ASD by setting up low-threshold offers that do not involve even more organizational challenges and stress for parents. In addition, parents should be given professional support for reflecting on their coping strategies in an attempt to then gradually reduce dysfunctional strategies for coping with parental demands. The aim here is to reduce or even prevent the buildup of stress.

## 5. Limitations

Using mediation analyses with cross-sectional data is controversial [e.g., (59)], so the results must be interpreted carefully. Nevertheless, the results can contribute to research when used to test existing causal theories. Wysocki et al. (60) demonstrate the importance of causal structures. In the present study, transactional stress theory (4, 5) serves as the causal structure to answer the research question using a mediation model.

It should be noted that the present sample size (*N* = 266) for the mediation analysis used, with a power level of 0.8 and a significance level of .05, can only identify effects above size .25. For smaller effects, according to the a-priori sample size calculator by Soper (61), a much larger sample would have been necessary (e.g., for anticipated effect size = .1, the minimum sample size to detect effect is *N* = 1.713). However, in order to achieve such sample sizes, larger, multi-centric projects are necessary in the future.

One limiting factor is that the data were collected via self-report by parents. Although anonymity was granted, it is unclear to what extent there is a social acceptability bias when parents are guided by how ideally stress should be handled (62). For example, questions about substance use (Substance use subscale) may make some individuals feel ashamed to state that they use alcohol or other substances (63). As mentioned above it is questionable whether other potential mediators should have been included in the model. The parental demands could have also been expanded, as only the parental demands in everyday life regarding the upbringing of the child with ASD were surveyed here. Although it is difficult to accurately cover everyday demands of different families because the realities of families' lives are heterogeneous, additional parental demands could have been included, such as household chores. In addition, the sample shows heterogeneity both because of the very wide age range of the children and because of the diagnosis and symptomatology of the children and adolescents with ASD. Future studies should focus on capturing the severity of ASD symptoms, as these may condition differences in parents' experience of stress [e.g., (64)]. Likewise, differences between mothers and fathers should have been considered, as studies show that mothers and fathers to children with ASD differ in their coping styles (40). In the present study, more than 85% of the parents interviewed were mothers, so the results cannot be interpreted in terms of gender without limitations. Not only should gender be taken into account, but more importantly, the amount of care work done by the parent being interviewed. It is likely that the demands on parents to children with ASD increased by life events like during the COVID-19 pandemic [e.g., higher caregiving demands due to quarantine; e.g., (65)]. Whether the present results will still be consistent then or whether different coping patterns will emerge to deal with the new demands remains to be investigated.

## Data Availability

The datasets presented in this article are not readily available because the data will be used for further analyses and publications. Requests to access the datasets should be directed to TS, teresa.sartor@tu-dortmund.de.
